# The Monoclonal Antibody Recognized the Open Reading Frame Protein in Porcine Circovirus Type 2-Infected Peripheral Blood Mononuclear Cells

**DOI:** 10.3390/v12090961

**Published:** 2020-08-29

**Authors:** Ling-Chu Hung

**Affiliations:** 1 Animal Health Research Institute, Council of Agriculture, Executive Yuan, New Taipei City 25158, Taiwan; lchung@mail.nvri.gov.tw; Tel.: +88-6226-212-111; 2 Livestock Research Institute, Council of Agriculture, Executive Yuan, Tainan 71246, Taiwan

**Keywords:** monoclonal antibody, open reading frame 3 protein, apoptosis, p53, porcine circovirus type 2, capsid protein, IgG, peripheral blood mononuclear cells, TUNEL, herd

## Abstract

The purpose of this study in the context of the open reading frame 3 (ORF3) protein of porcine circovirus type 2 (PCV2) was especially its location and its relation to the capsid protein and the apoptosis protein in PCV2-infected porcine peripheral blood mononuclear cells (PBMCs). To detect the ORF3 protein, monoclonal antibodies (mAbs) were generated in this study. The mAb 7D3 binds to the ORF3 peptide (residues 35–66) and the native ORF3 protein in PCV2-infected PBMCs, as shown by immunofluorescence assay (IFA). The data show that 3–5% of PBMCs were positive for ORF3 protein or p53 protein. Further, 78–82% of PBMCs were positive for the capsid. This study confirmed the ORF3 protein not only colocalized with the capsid protein but also colocalized with the p53 protein in PBMCs. Immunoassays were conducted in this study to detect the capsid protein, the ORF3 protein, anti-capsid IgG, and anti-ORF3 IgG. The data show the correlation (r = 0.758) of the ORF3 protein and the capsid protein in the blood samples from the PCV2-infected herd. However, each anti-viral protein IgG had a different curve of the profile in the same herd after vaccination. Overall, this study provides a blueprint to explore the ORF3 protein in PCV2-infected PBMCs.

## 1. Introduction

The Porcine circovirus (PCV) is a small virus and contains closed circular single-stranded DNA [1]. Previous studies indicated that porcine circovirus type 1 (PCV1) is non-pathogenic to porcine herd [2,3]. However, porcine circovirus type 2 (PCV2)-infected pigs showed dullness, thinness, lymphadenopathy, jaundice, hepatomegaly, and other manifestations [4,5,6,7,8]. Lymphocyte depletion and apoptosis of lymphocytes were the significant histopathological lesions in lymphoid tissues of PCV2-infected pigs [9,10]. PCV2 infection causes a reduction of T- and B-lymphocytes in the PBMC [11]. Likewise, PCV2-associated disease (PCVAD) manifested as severe swine herd problems [4,6,7,8]. Recently, PCVAD has become one of the significant swine diseases worldwide, and it impacts pork production [7]. At least four commercial PCV2a vaccines have been widely used to reduce viremia and PCV2 infectious pressure in swine herds [12,13,14,15]. That might have caused the global trend shift from PCV2a to PCV2b first [16,17,18,19], and then PCV2d (PCV2b mutant strain) has become a predominant genotype globally [20,21,22,23,24,25,26,27].

The open reading frame 1 (ORF1), open reading frame 2 (ORF2), and open reading frame 3 (ORF3) genes are the three principal ORFs among 11 ORFs in PCVs [28]. The ORF1 encodes for the replicase proteins Rep and Rep′ [28,29]. Rep protein represses the promoter of the *rep* gene by binding to the two inner hexamers H1 and H2. Although Rep′ binds to the same sequence, it does not influence Prep [30]. Notably, the ORF1 of PCV1 enhances the virus replication efficiencies of the chimeric viruses with the PCV2 backbone [31]. The primary structural capsid protein is encoded by ORF2 and has a molecular mass of 27.8–30 kDa [28,32]. Recently, the PCV2 mutant with the addition of the lysine residue in the capsid protein showed more virulence compared with classical PCV2a/b strains [33]. However, PCV2 is very different from PCV1, especially for the protein encoded by ORF3. The ORF3 protein in PCV2 is about half the size of its counterpart in PCV1 [28]. This protein plays a significant role in PCV2-induced apoptosis in porcine kidney cells (PK15) by activating initiator caspase-8 and effector caspase-3 pathways [34]. Further, the ORF3-deficient mutant PCV2 failed to induce any noticeable pathological lesions in animal experiments compared with the wild-type PCV2 [35,36].

Another study demonstrated that the ORF3 protein of PCV2 specifically interacted with the porcine p53-induced really interesting new gene (RING)-H2 (pPirh2) and inhibited its stabilization in the modulation of cellular function [37]. It had been demonstrated that the Pirh2 is a p53-induced ubiquitin-protein ligase and promotes p53 to degradation [38]. That is to say, the expression of Pirh2 decreases the level of p53 protein. On the other hand, the abrogation of Pirh2 results in a steady increase in p53 cellular levels. To put it another way, the ORF3 protein of PCV2 interacts with pPirh2 and facilitates p53 expression in PCV2 infection [37]. It is important to note that p53 plays a role in the regulation of the cell cycle, apoptosis, and genomic stability [39,40,41]. Researchers also indicated that the ORF3 protein competed with p53 in binding to pPirh2, and in particular the amino acid residues 20–50 or 35–65 of the ORF3 protein were essential in this competitive interaction of ORF3 protein with pPirh2 over p53 [42]. These events contribute to the deregulation of p53 by pPirh2, leading to increased p53 levels and apoptosis of the infected cells [37,42].

Some researchers indicated that ORF3 protein physically interacts with the host regulator of G protein signaling 16 (RGS16), leading to degradation of the RGS16 protein. Degeneration of RGS16 further enhances NF-κB translocation into the nucleus through the ERK1/2 signaling pathway and increases mRNA transcripts of the proinflammatory cytokines IL-6 and IL-8 [43]. Furthermore, the ORF3 protein-induced apoptosis aids in recruiting macrophages to phagocytize the infected apoptotic cells leading to the systemic dissemination of the infection [44]. As a result, the ORF3 protein is possibly the contributing factor for the pathogenesis and associated lesions [43,44]; however, there is little information about the ORF3 protein in the blood of PCV2-infected pigs. According to previous studies [34,37,42], the ORF3 protein has been characterized by using ORF3-transfected cells or PCV2-infected PK15 cells. The author raised the question of whether this protein exists in PCV2-infected apoptotic lymphocytes. To explore this question, the author decided to generate mAbs and antisera to label the ORF3 protein in this study.

For immunorelevant epitopes of the ORF3 protein, the ORF3 peptide (residues 31–50) was found to be the immunodominant T lymphocyte epitope [45]. Otherwise, residues 85–95 of the ORF3 protein suggested that this epitope might be masked in PCV2 infected cells [46,47]. The immunogenicity of the ORF3 peptide (residues 35–66) was demonstrated by PCV2-infected pig sera and the mouse immunological test [48]. Based on previous studies [45,48], there should be epitopes on the sequence of the ORF3 protein between residues 35 and 66. The other view is that the peptide (residues 35–65) of ORF3 protein should interact with the p53-binding domain of pPirh2 [42]. If the synthetic ORF3 peptide (residues 35–66) can mimic the epitopes of the native ORF3 protein, it could elicit antibodies that could bind to the native ORF3 protein.

Therefore, this study inoculated animals with the ORF3 peptide-containing immunogen to generate specific antibodies. This study generated two anti-ORF3 mAbs and defined their minimal binding regions. This study also used the anti-ORF3 mAb and other antibodies to label the ORF3 protein and p53 protein to determine apoptosis in PCV2-infected peripheral blood mononuclear cells (PBMCs). Furthermore, this study developed the antigen-ELISA to detect the ORF3 protein in the porcine blood from different age groups. The relationship among the ORF3 protein, the capsid protein, and their antibody responses in the PCV2-infected herd was also explored.

## 2. Materials and Methods

### 2.1. Design of Synthesized Peptides

Two peptides (C3 and N1) were described in the previous study [48]. Briefly, the peptide C3 contained the carboxyl-terminal (C-terminal) sequence of the capsid protein of PCV2b (GenBank: AAC35331.1) between residues 195 and 233, and the peptide N1 contained the sequence of the ORF3 protein of PCV2b (GenBank: AAC35332.1) between residues 35 and 66. The peptides (C3 and N1) were appended with an NH_2_-terminal (N-terminal) cysteine during synthesis, which was required for conjugation with the keyhole limpet hemocyanin (Thermo scientific, Rockford, IL, USA). Other peptides were synthesized by Yao-Hong Biotechnology Inc. (New Taipei, Taiwan), as listed in Table 1. These peptides contained the sequence of the ORF3 protein of PCV between residues 35 and 66, overlapping ten-mer peptides and associated peptides (20-mer, 22-mer, and 32-mer). Peptides P48 (PCV2a, GenBank: CBZ47005.1), P49 (PCV2, GenBank: AIA63974.1), and P56 (PCV1, GenBank: U49186) [28] were also used in this study. These truncated peptides were primarily used for the mapping of linear epitopes for anti-ORF3 mAbs binding. The purity of all peptides was assessed by high-performance liquid chromatography (LC-10ATVP serial dual plunger pump, Shimadzu, Torrance, CA, USA) and tested for the correct mass by Waters Micromass ZQ™ 2000 LC Mass Spectrometer (Waters, Milford, MA, USA).

### 2.2. Generation of Rabbit Antisera and MAbs against the ORF3 Peptide (Residues 35–66))

Animal experiments in this study were performed following the current legislation on ethical and welfare recommendations. The experiments followed the standards of the Guide of the Care and Use of Laboratory Animals. All animal work was approved by the Institutional Animal Care and Use Committee (IACUC) of the Livestock Research Institute, and the IACUC of the Animal Health Research Institute. The IACUC approval numbers LRIIACUC100-33 (for swine and murine experiments), A00027 (for murine and rabbit experiments), and A02023 (for murine and rabbit experiments) were given in this study.

Antisera against PCV2 were generated by immunizing New Zealand white rabbits (from Livestock Research Institute, Council of Agriculture, Taiwan). Rabbits were maintained in isolation rooms, and the room temperature was at 20–26 °C. They were fed with a commercially pelleted diet (Fwusow In., Taiwan), and pure water was available ad libitum. Then, animals were immunized with peptide immunogen (the peptide N1 conjugated with KLH) or commercial vaccine five times at two-week intervals. For peptide immunization, an initial dose of 150 μg of the conjugated peptide was mixed with complete Freund’s adjuvant (Sigma-Aldrich, St. Louis, MO, USA) at the first injection.

Each rabbit was re-immunized with the same amount of immunogenic mixture with incomplete Freund′s adjuvant (Sigma-Aldrich, St. Louis, MO, USA). For virus-like particles (VLP) of PCV2 immunization, the rabbit was injected intramuscularly in the legs with 0.5 mL of the PCV2 vaccine (CircoFLEX^®^, Boehringer Ingelheim). The blood was harvested two weeks after the last injection. The antibody titers were measured by indirect enzyme-linked immunosorbent assay (iELISA) [48] (performed as described previously for mouse anti-PCV2 ELISA, with the exception that peroxidase-conjugated donkey anti-rabbit IgG (H + L, Jackson ImmunoResearch, West Grove, PA, USA) secondary antibody was used).

Hybridomas were generated following established methods, as previously described [49]. Briefly, four five-week-old, female, BALB/cByJNarl mice were purchased from a specific pathogen-free (SPF) colony (the National Applied Research Laboratories, Taiwan). The mice were maintained in isolation rooms in filtertop cages, and the room temperature was at 20–26 °C. Mice were fed with a commercially pelleted diet (LabDiet, 5002, St. Louis, MO, USA), and pure water was available ad libitum. Mice were inoculated with 65 μg of immunogen (the peptide N1 conjugated with KLH) emulsified in Freund’s adjuvant. Subsequently, mice were boosted fortnightly with the same amount of immunogen. Three days after the final booster, the spleens of the mice were harvested for hybridoma generation. Hybridomas were screened for the secretion of anti-ORF3-specific mAbs by iELISA [49] using the peptide N1 as a coating antigen. That secondary antibody solution (HRP-conjugated goat anti-mouse IgA/IgG/IgM, H/L chain (Novus, Saint Charles, MO, USA) was used at 1:2500 dilution for hybridoma screening. The hybridomas that secreted anti-N1 mAbs were subcloned by limited dilution of the cells.

Then, ascites containing mAbs were collected as previously described [49]. Subsequently, the mAbs were purified from the ascites by using the NAb Protein G Spin Column (Thermo Scientific, Rockford, IL, USA), then collecting solutions were exchanged for the buffer and concentrated using the Amicon Ultra 50K (Millipore, Burlington, MA, USA). The concentration of mAbs was determined using the Nano Photometer^®^ (Implen, Munich, Germany).

### 2.3. Isotyping of MAbs

The heavy chain and the light chain of the two mAbs secreted by each hybridoma were determined by using an SBA Clonotyping System/HRP (Southern Biotechnology, Birmingham, AL, USA). It was performed according to the manufacturer’s instructions and a previously published method [49]. Briefly, the Nunc MaxiSorp flat-bottom 96-well plate (Thermo Fisher Scientific, Inc., Waltham, MA, USA) was coated with the capture antibody (goat anti-mouse Ig, 5 μg/mL) in 0.05-M bicarbonate buffer (Sigma-Aldrich, St. Louis, MO, USA) at 4 °C overnight. After three washes with PBS containing 0.05% Tween 20 (PBST), the plate was blocked with the blocking buffer (PBST containing 5% casein hydrolysate) at 37 °C for 30 min. After washing, 100 μL of each culture supernatant of hybridoma were added sequentially, and the plate was incubated at 37 °C for 2 h. After washing, 100 μL of HRP labeled goat anti-mouse IgG1, -IgG2a, -IgG2b, -IgG3, -IgA, -IgM, -κ light chain, and -λ light chain were added to appropriate wells of the plate. PBST served as background. The plate was then incubated at 37 °C for 1 h and after that washed with PBST. Finally, 100 μL of 2, 2′-azino-bis (3-ethylbenzothiazoline-6-sulfonic acid) diammonium salt (ABTS, Sigma-Aldrich, St. Louis, MO, USA) buffer were added to each well of the plate. After 30 min, the optical density (OD) of each well was measured at 405 nm using a SpectraMax M5 microplate reader (Molecular Devices, San Jose, CA, USA).

### 2.4. Epitope Mapping

The epitope mapping of these mAbs was performed by iELISA [49] using truncated peptides (as shown in Table 1) as coating antigens. Briefly, peptides contained the sequence of ORF3 protein of PCV2b between residues 35 and 66, associated 10-mer peptides, truncated derivatives, and the control (peptide C3). Ninety-six-well Maxisorp plates were coated with each peptide (5 μg/mL) in bicarbonate buffer and incubated at 4 °C overnight. After three washes in PBST, the plates were blocked with the blocking buffer at 37 °C for 30 min. After washing, the culture supernatant of hybridoma was added, and plates were again incubated at 37 °C for 2 h. The anti-peptide N1 mouse serum (at a 1:1000 dilution) was used as the positive control, and the anti-ORF2 (peptide C3) mAb (1H3) was served as the negative control. After rinsing three times with PBST, the secondary antibody solution was applied to the appropriate wells. Peroxidase-conjugated goat anti-mouse IgG (subclasses 1 + 2a + 2b + 3, Fcγ, Jackson ImmunoResearch, West Grove, PA, USA) was used at a 1:2500 dilution for binding to the mAb 7D3. HRP-conjugated goat anti-mouse lambda light chain (Novus, Saint Charles, MO, USA) was used at a 1:5000 dilution for binding to the mAb 6D10. After 1 h, the plates were washed three times. The colorimetric reaction was developed by using the ABTS solution. Following a 30-min incubation, the OD was measured at 405 nm.

### 2.5. Western Blot Analyses Determined the Molecular Weight of MAb 6D10

Both of the isotype and molecular weight of the mAb 6D10 were determined by Western blotting. The culture supernatant of the hybridoma (clone 6D10) was separated by BoltTM Bis-Tris Plus Gel (Invitrogen, Carlsbad, CA, USA). One gel was stained with FaStain Protein Staining Solution (Yeastern Biotech, Taiwan). The other gel was transferred to the polyvinylidene difluoride (PVDF) membranes (Millipore, Burlington, MA, USA) with Fast Semi-Dry transfer buffer (Thermo, Waltham, MA, USA) by using a Yrdimes Semi-dry transfer system (Wealtec, New Taipei City, Taiwan). The membrane was blocked with the blocking buffer for 30 min at 37 °C.

After three washes in PBST, each strip of the membrane was incubated with one appropriate horseradish peroxidase (HRP)-conjugated antibody to detect mAb at 4 °C overnight and then at room temperature for 2 h. Peroxidase-conjugated goat anti-mouse IgA/IgG/IgM, H/L chain (Novus, Saint Charles, MO, USA) at 1:2500, HRP-conjugated goat anti-mouse IgA (Southern Biotechnology, Birmingham, AL, USA) at 1:2500, HRP-conjugated goat anti-mouse lambda light chain (Novus, Saint Charles, MO, USA) at 1:5000, HRP-conjugated goat anti-mouse IgM, μ chain (Jackson Immunoresearch, West Grove, PA, USA) at 1:5000, and HRP-conjugated goat anti-mouse IgG (subclasses 1 + 2a + 2b + 3, Fcγ, Jackson Immunoresearch, West Grove, PA, USA) at 1:2500 were used in this assay. Then, they were washed three times with PBST, 5 min each wash. Each strip was incubated with 3, 3′-diaminobenzidine (DAB, horseradish peroxidase substrate, Millipore, Burlington, MA, USA) buffer for 5 min. Protein molecular weight markers were included in each Western blot analysis. After revelation with the DAB substrate, the target protein was visualized and calculated the molecular weight by using molecular weight markers.

### 2.6. Immunofluorescence Assay of PCV2-Infected PBMCs

Homemade immunofluorescence assay (IFA) slides were performed as previously described [49]. PCV2-infected peripheral blood mononuclear cells (PBMCs) were collected from the PCV2-infected conventional piglet. The pig serum was detected as seropositive for PCV2 by anti-capsid peptide (C3) specific immunoassay [48]. The whole blood sample was confirmed as positive for PCV2 antigen by using the INGEZIM PCV DAS kit (Ingenasa, Madrid, Spain). Briefly, PBMCs were isolated from ethylenediaminetetraacetic acid (EDTA)-treated whole blood samples by Ficoll-Paque Plus (GE Healthcare Bio-Sciences, Uppsala, Sweden) and according to the manufacturer’s instructions. Then, mononuclear cells were drawn from the interface of the Ficoll-Paque Plus-containing tube and resuspended in 45 mL PBS for washing and centrifugation (100× *g* for 10 min at 4 °C). After washing three times with PBS, the cell pellet was resuspended in 0.5 mL of fetal bovine serum. An aliquot (25 μL) of cell suspension was loaded onto a glass slide for the cell smear. After the cell smear, the slides were air-dried at room temperature. Thereby, the PBMCs were stuck on slides. These slides were fixed with PBS containing 2% paraformaldehyde at 4 °C for 10 min and then washed three times with PBS. Following that, the slides were soaked in PBS containing 0.1% Triton X-100 at 4 °C for 3 min. Subsequently, slides were washed with PBS and prepared for IFA.

Two anti-capsid (the peptide C3) mAbs (1H3 and 6B8) [49], one anti-PCV2 mAb 36A9 (Ingenasa, Madrid, Spain), one anti-p53 protein (wt-p53) rabbit polyclonal antibody (Bioss, Woburn, MA, USA), the homemade anti-ORF3 peptide (N1) mAb (7D3), and rabbit antisera (as the method mentioned above) were used in this study. The anti-capsid peptide mAbs (1H3 and 6B8) recognized native capsid proteins and reacted with core peptides (P59, _227_KDPPLNP_233_); however, the mAb 1H3 bound the three minimal linear epitopes (DPPLNP, DPPLNPK, and LKDPPLKP) and the mAb 6B8 bound two minimal linear epitopes (KDPPLNP and KDPPLNPK). These mAbs produced a variable definite staining pattern in PBMCs by IFA [49].

The PCV2-infected PBMCs slides were incubated with a 1:100 dilution of rabbit antiserum and a 1:100 dilution of mAb (ascites). After incubation at 37 °C for 1 h, the slides were gently rinsed briefly in PBS and then soaked for 15 min in PBS at 4 °C. The slides were then incubated at 37 °C with TRITC-conjugated goat anti-mouse IgG (subclasses 1 + 2a + 2b + 3, Fcγ) at a 1:100 dilution and FITC-conjugated goat anti-rabbit IgG (H + L) (all from Jackson ImmunoResearch, West Grove, PA, USA, and each minimal antibody cross-reaction to other animal’s serum protein) at a 1:100 dilution. After 30 min of incubation, the slides were washed with PBS and then incubated with 4, 6-Diamidino-2-phenylindole, dihydrochloride (DAPI, AAT Bioquest, Sunnyvale, CA, USA) at a 1:2300 dilution in PBS at room temperature for 15 min. The slides were mounted under 50% glycerol and observed with an Olympus BX51 fluorescence microscope and SPOT Flex camera (Diagnostic Instrument, Model 15.2 64MP, USA).

### 2.7. TUNEL Assay of PCV2-Infected PBMCs

Terminal deoxynucleotidyl transferase dUTP nick end labeling (TUNEL) assay was performed according to the manufacturer’s instructions (The Cell Meter™ TUNEL Apoptosis Assay kit, AAT Bioquest^®^, Inc., Sunnyvale, CA, USA) to detect excessive DNA breakage in the PBMC. Briefly, the PCV2-infected PBMCs slides were incubated with the TF3-dUTP/reaction buffer. After 60 min of incubation, the slides were washed with PBS. They were then incubated with Hoechst solution at room temperature for 13 min. The slides were mounted under 50% glycerol and observed with an Olympus BX51 fluorescence microscope and SPOT Flex camera.

### 2.8. Detection of the Capsid Protein and the ORF3 Protein in the Whole-Blood Samples from the PCV2-Infected Herd with a PCV2 Vaccine

Blood samples were collected from the PCV2-infected herd with the following vaccination at two weeks of age with a PCV2 vaccine (2 mL, Porcilis^®^ PCV, MSD Animal Health) in the conventional pig farm with the farrow-to-finish operation. Pigs were selected from animals of different ages (1, 3, 6, 9, 12, 15, 18, 21, 24, and ≥34 weeks of age) at one time. Then, four samples were randomly chosen per age group. Blood was collected in the Vacutainer^®^ EDTA tubes (Becton and Dickinson BV) and sored at −20 °C. All whole-blood samples had been treated by the freeze–thaw process twice before the test.

The capsid protein was detected from 50 μL of whole-blood sample by using the INGEZIM PCV DAS kit (Ingenasa, Madrid, Spain) according to the manufacturer’s instructions, and the absorbance of each well was measured at 450 nm. Meanwhile, the homemade antigen-ELISA was conducted in this study for detecting the ORF3 protein in whole-blood specimens. It was based on the double-antibody-sandwich principle. Ninety-six-well Maxisorp plates were coated with 1 μL/mL (100 μL per well) of the trapping sera (rabbit anti-ORF3 peptide) in bicarbonate buffer. The plates were incubated at 4 °C overnight and washed three times with PBST. Then, the plates were blocked with the blocking buffer at 37 °C for 30 min. After the plates were washed, 50 μL of PBST and 50 μL of whole-blood samples were mixed and added to the well. The plates were incubated at 37 °C for 2 h and then washed in PBST. Then, 100 μL/well of mAb 7D3 at 1 μg/mL were added to the plates, and the plates were incubated at 37 °C for 1 h. After the plates were washed, 100 μL/well of peroxidase-conjugated goat anti-mouse IgG Fcγ at a 1:2500 dilution were added, and the plates were incubated at 37 °C for 1 h. After washing, ABTS buffer was added to each well. Following a 30-min incubation, the OD was measured at 405 nm.

### 2.9. Detection of the Specific Antibodies Against the Capsid Protein or the ORF3 Protein in Plasm Samples from the PCV2-Infected Herd with a PCV2 Vaccine

Plasm samples were separated from the aforementioned fresh whole-blood samples and sored at−20 °C. The antibody titers were measured by iELISA [48] (performed as described previously for pig anti-peptide ELISA, with the exception that the capsid protein (CircoFLEX^®^, Boehringer Ingelheim) or peptide P126 was used as the coating antigen, and pig plasm at a 1:200 dilution was used). The subsequent procedures of blocking, pig antibody, washing, secondary antibody, substrate, and the OD reading, were the same as described previously.

### 2.10. Statistical Analysis

The data were analyzed by using one-way analysis of variance (ANOVA) and Tukey’s Studentized Range (HSD, honestly significant difference) multiple comparisons test using the SAS Enterprise Guide 7.1^®^ software (SAS Institute Inc., Cary, NC, USA). A *p*-value < 0.05 was considered significant.

## 3. Results

### 3.1. Rabbit Antisera Against the PCV2 Peptides

The ORF3 peptide N1 or VLP of PCV2 was capable of inducing each specific antibody. The antibody titer of each post-immune serum (two weeks after the fourth booster) was detected at the dilution 1:10000 (Figure S1). The binding of the rabbit antiserum to the virus was confirmed by using an immunofluorescence assay (IFA) (Figure S2). Although pre-immune serum had a low background (OD_405_ value less than 0.4) at a dilution of 1:100, the pre-immune serum did not bind the virus as detected by IFA (data not shown). The author checked for the cross-reactivity of post-immune antiserum to other antigens. The result shows they have a minor cross-reaction at a dilution of 1:100 (Figure S1), but the OD_405_ value of cross-reaction has not significantly higher than that of the negative control serum (*p* ≥ 0.05) (Figure S1).

### 3.2. Hybridomas Screening and Isotyping of Anti-ORF3 (N1) MAbs

**Figure 1:**
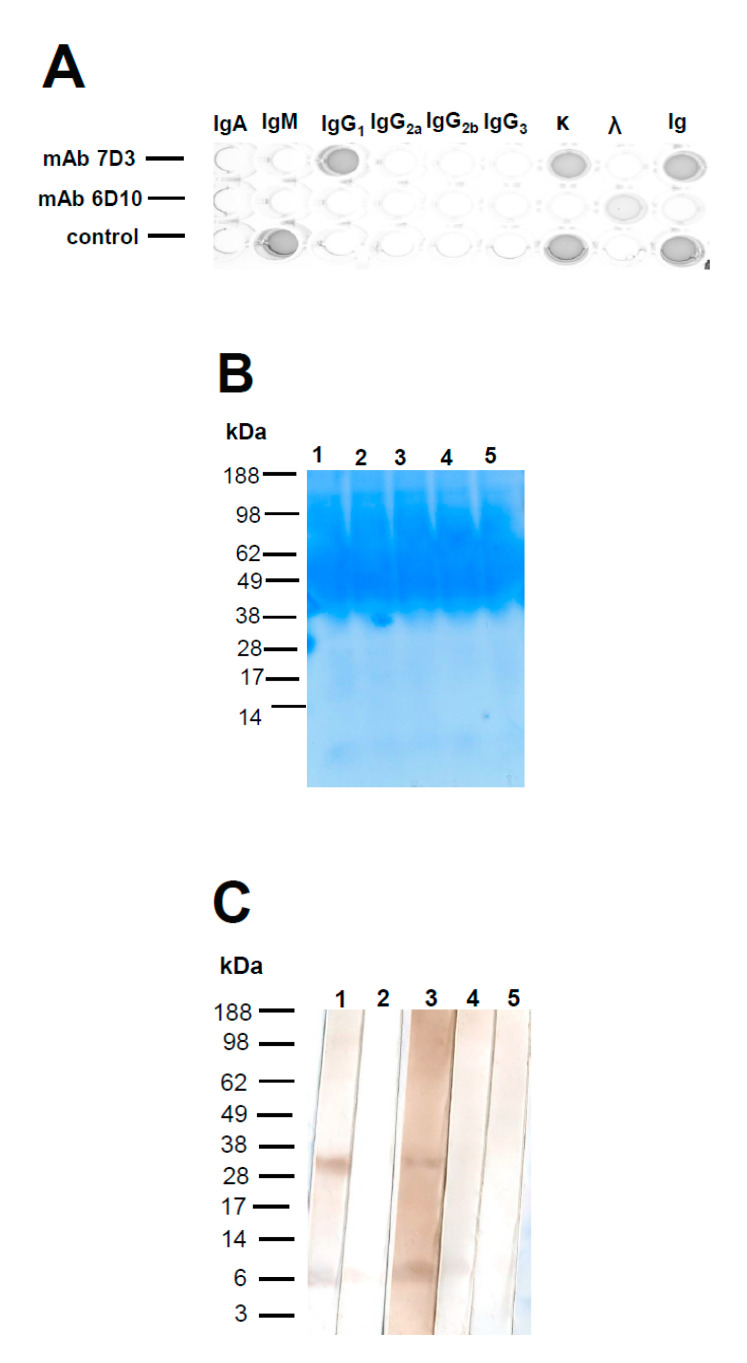
Isotyping of anti-open reading frame 3 (ORF3) mAbs. Isotyping was determined by using an SBA Clonotyping System/HRP (**A**). (top row) mAb 7D3; (middle row) mAb 6D10; and (bottom row) control sample (IgM). IgA, IgM, IgG1, IgG2a, IgG2b, IgG3, κ light chain, λ light chain, and Ig were examined on each appropriate column as shown. The culture supernatant of a hybridoma (clone 6D10) was separated by BoltTM Bis-Tris Plus Gel (**B**). The molecular weight and isotype of the mAb 6D10 were determined by Western blotting (**C**). Strip 1 was stained with a peroxidase-conjugated goat anti-mouse IgA/IgG/IgM, H/L chain to identify all kinds of immunoglobulins. Strip 2 was stained with an HRP-conjugated goat anti-mouse Ig A (α chain). Strip 3 was stained with an HRP-conjugated goat anti-mouse lambda light chain. Strip 4 was stained with an HRP-conjugated goat anti-mouse IgM (μ chain). Strip 5 was stained with HRP-conjugated goat anti-mouse IgG (Fcγ).

Following the fusions, hybridoma supernatants were screened for the presence of anti-ORF3 (peptide N1) specific antibodies by iELISA. Among them, 34 hybridomas supernatants reacted with the peptide N1 at the first screening (data not shown). After repeatedly subcloning by the limiting dilution and selection, two stable hybridomas secreting anti-ORF3 mAbs (7D3 and 6D10) were obtained. The hybridoma produced IgG1 mAb (7D3) with kappa-light chains. The other produced mAb (6D10) with a lambda-light chain (Figure 1A); however, no heavy chain was detected in the supernatant of clone 6D10 by SBA Clonotyping System/HRP assay. The supernatant of a hybridoma (clone 6D10) was separated directly by BoltTM Bis-Tris Plus Gel to confirm the heavy chain of this mAb. The gel showed a broad staining region within 40–98 kDa. The author speculated that the supernatant of a hybridoma contains abundant serum proteins from the fetal bovine serum-containing medium. That is to say, the supernatant of a hybridoma (clone 6D10) contains a little mAb 6D10 (light chain). Further, the Western blotting was used to confirm the heavy chain of mAb 6D10 and its molecular weight. The HRP-conjugated antibodies (goat anti-mouse IgA/IgG/IgM, H/L chain, and goat anti-mouse lambda light chain) gave specific reactions with the supernatant. Two bands were observed at about 33 (between 28 and 38) and 6 kDa, respectively (Figure 1). The lower band (6 kDa) implied that some degradation of this mAb. However, other anti-Ig antibodies (goat anti-mouse Ig A (α chain), goat anti-mouse IgM (μ chain), and goat anti-mouse IgG (Fcγ)) gave negative reactions with the supernatant. This study confirmed mAb 6D10 contains a lambda light chain, but it does not include any intact heavy chain. Since the molecular weight of the light chain was 25 kDa, it may be assumed that the mAb 6D10 might contain a short fragment of a protein.

### 3.3. Mapping of Linear Epitopes for Anti-ORF3 MAbs Binding

**Figure 2:**
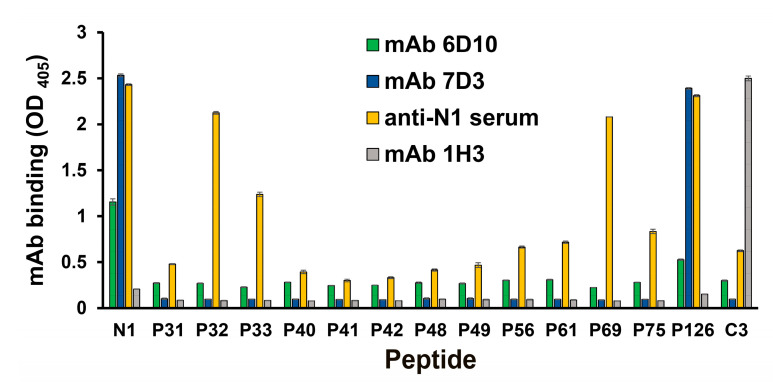
Mapping of the linear and minimal epitopes for anti-ORF3 (peptide N1) mAbs binding. Anti-ORF3 mAbs (7D3 and 6D10) bound the linear peptide spanning from residues 35 to 66. The anti-peptide N1 mouse serum was used as the positive control, and the anti-ORF2 (peptide C3) mAb (1H3) served as the negative control. The mAb binding was tested by using an iELISA. Peptides contained the sequence of the ORF3 protein between residues 35 and 66, associated 10-mer peptides, truncated derivatives, and the control (peptide C3) (as shown in Table 1). The experiments were performed three times. Data represent the mean ± standard error (SE).

A peptide scan analysis was performed to determine the binding site for each mAb (Figure 2). The mAb 7D3 bound to two linear peptides (peptide N1 and P126). The peptide N1 was appended with a cysteine residue at the N-terminal of the ORF3 peptide (residues 35–65, P126). Both of them contained the sequence of the ORF3 protein of PCV2b between residues 35 and 66. However, the non-IgG mAb (6D10) not only reacted with peptide N1 but also showed minor reactions against other peptides, which compared with the mAb 7D3 in the ELISA test (Figure 2). Interestingly, the anti-peptide N1 mouse serum reacted all test peptides and bound firmly to six linear peptides (peptide N1, P32, P33, P69, and P126). Notably, anti-N1 poly antibodies reacted with these peptides (expect P33) which contained the common core peptide (P32 and _45_TLLHFPAHFQ_54_). Nevertheless, this study did not get any mAb which bound firmly to the peptide P32.

### 3.4. The ORF3 Protein Colocalized with the Capsid Protein or the P53 Protein in Some PCV2-Infected PBMCs

For these homemade PBMCs slides, all slides were made from the same batch and contained the same proportion of PCV2-infected cells. All wells contained both negative and positive cells (PCV2-infected cells) confirmed by IFA, as previously noted in a similar study [49].

**Figure 3:**
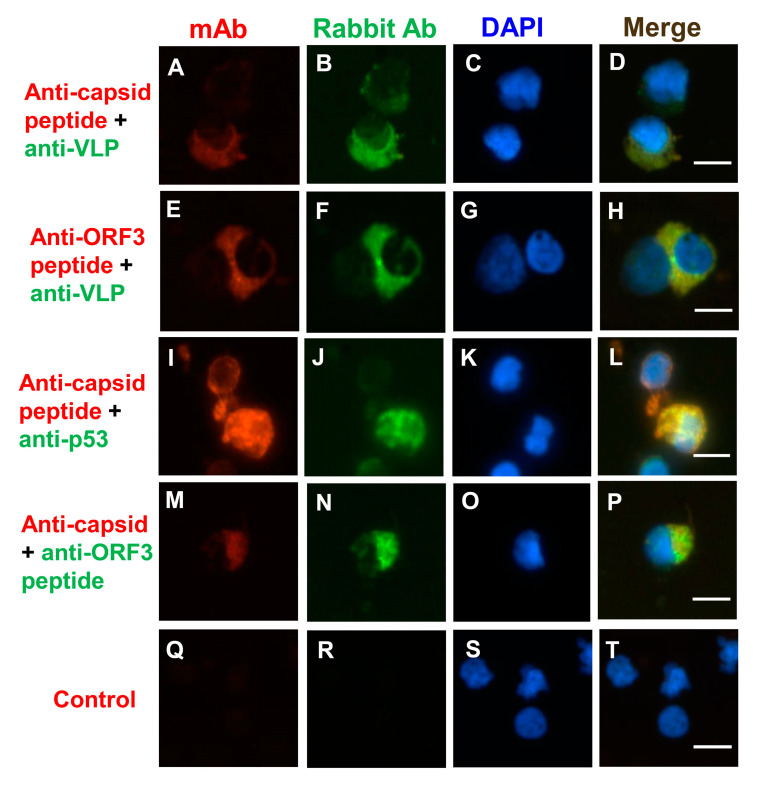

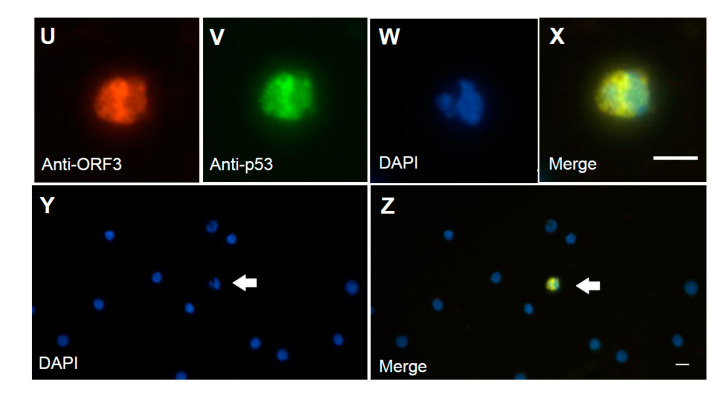
The localization of viral proteins of PCV2 on PCV2-infected peripheral blood mononuclear cells (PBMCs). The localization of viral proteins of PCV2 was assessed by indirect immunofluorescence assay (IFA). The positive signals were due to different abundance of the protein, the degradation of the protein, different PCV2 strains, conformational epitopes, or linear epitopes, which were interaction with the antibody. This study used rabbit antiserum (green) and co-stained with mouse mAbs (red) to mark various viral epitopes. Anti-capsid peptide mAb 6B8 bound two minimal linear epitopes (KDPPLNP and KDPPLNPK), and anti-PCV2 capsid peptide mAb 1H3 bound the three minimal linear epitopes (DPPLNP, DPPLNPK, and LKDPPLKP). Anti-ORF3 peptide mAb 7D3 bound the minimal linear epitope (HNDVYISLPITLLHFPAHFQKFSQPAEISDKR). (**A**,**E**,**I**,**M**,**U**) Staining with mouse mAbs (red) was used to identify the capsid protein of PCV2 (mAb 6B8 (**A**); mAb 1H3 (**I**); and mAb 36A9 (**M**)), and the ORF3 peptide of PCV2 (mAb7D3 (**E**,**U**)). Staining with SPF mouse serum was used as the negative control (**Q**). (**B**,**F**,**J**,**N**,**V**) Staining with rabbit antiserum (green) was used to identify the VLP of PCV2 (**B**,**F**), the p53 (**J**,**V**), and the ORF3 peptide of PCV2 (**N**). (**C**,**G**,**K,O**,**S**,**W**,**Y**) Nuclei were stained with DAPI (blue). The arrow points to the positive staining cell (**Z**) and its irregular shaped nucleus (**Y**), and the enlarged image in the sixth row (**X**,**W**). (**D**,**H**,**L**,**P**,**T**,**X**,**Z**) The merged images are also shown. The scale bar is 10 μm.

In this study, PCV2 viral protein (ORF3 protein and capsid protein) locations were initially assessed by IFA. The previous study indicated that the mAb 1H3 bound the three minimal linear epitopes (DPPLNP, DPPLNPK, and LKDPPLKP), which were located at carboxyl-terminus (Cterminus) of the capsid proteins of PCV2b, PCV2d, and PCV2a, respectively [49]. Likewise, the mAb 6B8 bound two minimal linear epitopes (KDPPLNP and KDPPLNPK), which were located at Cterminus of the capsid proteins of PCV2b, and PCV2d, respectively [49]. First, the percentage was determined by counting the number of positive cells per 100 nuclei in an IFA slide. The data show that 78–82% of PBMCs were positive for anti-capsid peptide mAb (1H3) staining (PCV2a-, PCV2b-, or PCV2d-infected PBMCs), 48–51% of PBMCs were positive for anti-capsid peptide mAb (6B8) staining (PCV2b- or PCV2d-infected PBMCs), 33–38% of PBMCs were positive for anti-VLP rabbit serum staining (PCV2a-infected PBMCs), 4–5% of PBMCs were positive for anti-capsid mAb (36A9) staining (PCV2a-infected PBMCs), and 3–4% of PBMCs were positive for anti-ORF3 mAb (7D3) staining (Figure S3). It might be due to different abundance of the protein, the degradation of the protein, different PCV2 strains, conformational epitopes, or linear epitopes, which interacted with the antibody. Interestingly, the anti-VLP of PCV2 rabbit serum produced cytoplasmic staining in PCV2-infected PBMCs (Figure 3B,F). When PCV2-infected PBMCs were co-stained with anti-VLP of PCV2 rabbit serum and anti-capsid peptide mAbs (6B8), some VLP/capsid peptide dual-positive cells were shown (Figure 3D). The overlap between the anti-VLP rabbit serum staining and the anti-ORF3 mAb (7D3) staining is presented in Figure 3H, and the anti-ORF3 polyclonal antibody and anti-capsid mAb (36A9) overlapped the same in Figure 3P; however, the polyclonal antibody staining was brighter than the mAb staining. This result shows ORF3 protein overlaps the capsid protein (or VLP) of PCV2 (Figure 3H,P). Both the capsid proteins and the ORF3 protein were detected mainly in the cytoplasm. According to the previous study, the N-terminal half of ORF3 protein (residues 1–53) was discovered in the cytoplasm, and the C-terminal half of ORF3 protein (residues 53–104) was majorly located in the nucleus [50]. The medial region of ORF3 protein (residues 35–66) might be primarily located in the cytoplasm in this study.

**Figure 4:**
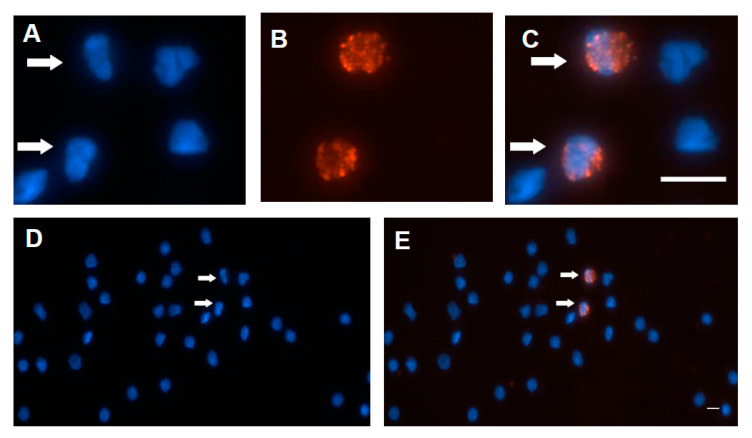
Detecting excessive DNA breakage in PCV2-infected PBMCs. The Cell Meter™ TUNEL Apoptosis Assay kit was performed. At the same time, the percentage was determined by counting the number of positive cells per 100 nuclei in a slide. The data show that 3–4% PBMCs were positive for TUNEL staining. (**A**,**D**) Nuclei were stained with Hoechst. The arrow points to the cashew-shaped nucleus (**D**), and the enlarged image is also shown (**A**). Staining with TF3-dUTP/reaction buffer (red) was used to detect DNA fragmentation (**B**). The merged images are also shown (**C**,**E**). The scale bar is 10 μm.

A previous study suggested that ORF3 protein led to increased p53 protein levels and apoptosis of the infected cells [42]. To identify the subcellular location of the ORF3 protein in PCV2-infected PBMCs, anti-capsid, anti-ORF3, and anti-p53 antibodies were used. The p53 protein also colocalized with capsid peptide (Figure 3L), and the ORF3 peptide (Figure 3X). The p53 protein was mainly distributed in the cytoplasm and around the nucleus (Figure 3J,V). The ORF3 protein, as detected with mAb 7D3, seemed to colocalize quite nicely with the p53 protein (Figure 3X). The p53/ORF3 dual-positive cell presented a segmented nucleus (Figure 3W,Y). The nuclei of negative cells were round or oval, and nuclear segmentation was very rare in negative cells (Figure 3Y). The apoptosis in PCV2-infected PBMCs was observed by using a terminal deoxynucleotidyl transferase dUTP nick end labeling (TUNEL) assay (Figure 4 and Figure S4). The data show that 3–4% PBMCs were positive for TUNEL staining. It is very similar to the percentage of anti-ORF3 mAb (7D3) staining. However, these similar percentages are not sufficient proof to show that the ORF3 protein of PCV2 causes DNA damage. A more careful analysis would be needed to reveal whether PCV2 infection causes DNA damage. Unfortunately, it was not easy to label both the ORF3 protein and the signals of DNA breakage simultaneously by IFA and TUNEL assay (data not shown). Further research would be needed to determine whether the ORF3 protein colocalizes with the signals of TUNEL.

### 3.5. The ORF3 Protein was Correlated with the Capsid Protein in the Whole-Blood Samples

**Figure 5:**
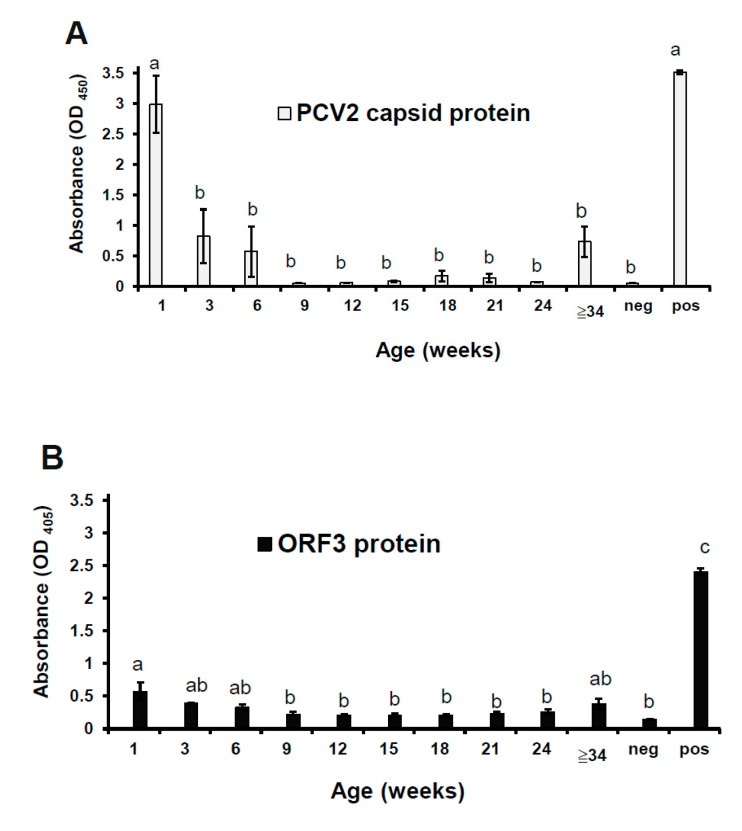
Assessment of the capsid protein and the ORF3 protein in the whole-blood samples from pigs of different age groups. This study used the commercial capsid kit (**A**) and the ORF3 protein ELISA (**B**) to measure each specimen, and the absorbance was measured at 450 or 405 nm, respectively. The peptide N1 and PSBT were used as a positive (pos) control and a negative (neg) control, respectively, in the ORF3 protein ELISA. The data represent the mean ± SE. Treatments with different letters have statistically significant differences (*p* < 0.05).

The viral proteins were detected in the whole-blood samples using antigen-ELISA techniques. The cut-off value in the commercial PCV2 capsid ELISA and the ORF3 protein ELISA were 0.15 and 0.22, respectively. The capsid protein of PCV2 was mainly detected in pigs at 1, 3, 6, and ≥34 weeks of age in this study farm (Figure 5A). Noticeably, these groups (at 1, 3, 6, and ≥34 weeks of age) showed more elevated standard error of OD_405_ value than other groups in this statistical analysis. This result implies that pigs at that age would be susceptible to PCV2 infection. Interestingly, non-vaccinated piglets showed the highest OD_450_ value of capsid protein at one week of age. After piglets were inoculated with the PCV2 vaccine at two weeks of age, the OD_450_ value of capsid protein gradually decreased until 24 weeks of age. However, the OD_450_ value of capsid protein was raised at ≥34 weeks of age (two gilts and two sows). There is evidence to suggest that the vaccine could not protect pigs against PCV2 infection at 32 weeks postimmunization. The ORF3 protein of PCV2 showed the same result except for OD values of the ORF3 protein were lower than the capsid protein. The correlation coefficient (*r*) was 0.7583. The mean OD_405_ values of the ORF3 protein were significantly higher for suckers at one week of age than postweaning ones at 9–24 weeks of age (*p* < 0.05; Figure 5B). There is an apparent probability that the amount of ORF3 protein was fewer than the amount of capsid protein in PCV2-infected blood from suckers at one week of age.

### 3.6. The Anti-ORF3 Peptide IgG and Anti-Capsid IgG Response in the Pig Herd

**Figure 6:**
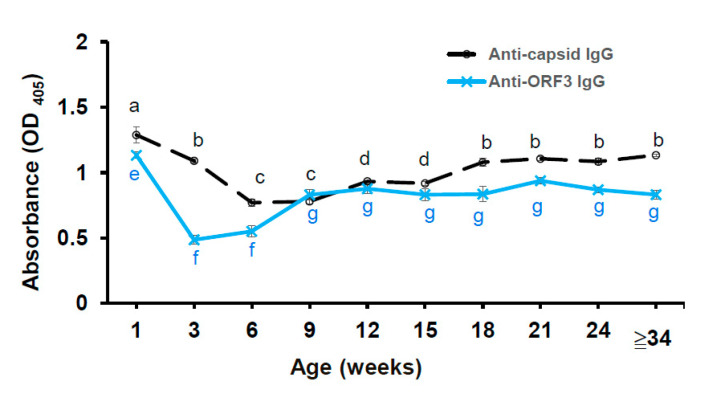
Assessment of the anti-capsid IgG and the anti-ORF3 peptide (P126) in the plasm samples from pigs of different age groups. The experiments were repeated three times, and data represent the mean ± SE. Treatments with different letters at the same kind of antibody assay have statistically significant differences (*p* < 0.05).

The anti-viral protein-specific antibodies were detected in the plasm samples using iELISA techniques. The cut-off value of in the anti-capsid IgG ELISA and the anti-ORF3 peptide (P126) IgG ELISA were 0.20 and 0.18, respectively. Non-vaccinated suckers showed the highest mean OD_405_ value of anti-capsid IgG and anti-ORF3 IgG at one week of age. It may mean that non-vaccinated suckers absorbed large amounts of maternally derived antibodies after birth. Notably, the non-vaccinated group showed the most elevated standard error of OD_405_ value (anti-capsid IgG) in this statistical analysis. A gradual decrease of the mean OD_405_ value of anti-capsid IgG for piglets from the suckling period (one week of age) to the weaning period (six weeks of age) was observed. From 9 to 12 weeks old, the mean OD_405_ value of anti-capsid IgG increased significantly (*p* < 0.05; Figure 6). The mean OD_405_ value of anti-capsid IgG also increased significantly for pigs from 15 to 18 weeks old (*p* < 0.05) and reached a plateau at 18 weeks old. The same trend was observed in the result of the anti-ORF3 IgG ELISA test, except for OD_405_ values of anti-ORF3 IgG were lower than anti-capsid IgG. Notwithstanding, it is worth mentioning that there was a sharp decrease of the mean OD_405_ value of anti-ORF3 IgG for suckers from one to three weeks old. The mean OD_405_ value of anti-ORF3 IgG increased significantly for pigs from six to nine weeks old (*p* < 0.05; Figure 6) and reached a plateau at nine weeks old. There was a weak correlation (*r* = 0.3771) between the OD_405_ value of anti-capsid IgG and the OD_405_ value of anti-ORF3 IgG in the plasm samples. It points to the probability that the amount of anti-capsid IgG reflects humoral immunity in PCV2-infected pigs before and after the injection of a PCV2 vaccine. Besides, the amount of anti-ORF3 IgG reflects humoral immunity in pigs, which were caused by PCV2 infecting only.

## 4. Discussion

According to a previous study, the ORF3 peptide (residues 35–65) of PCV2 interacts with the p53-binding domain of pPirh2 [42]. The peptide N1 (residues 35–66) of the ORF3 protein was synthesized and used in this study. Subsequently, anti-ORF3 mAbs were generated and characterized in this study. This work created one hybridoma producing the mAb 7D3 with IgG1, and the other producing the defective-Ig mAb (6D10) with a lambda-light chain. The mAb 7D3 (IgG1) bound to the linear peptide N1 (C_35_HNDVYISLPI TLLHFPAHFQ KFSQPAEISDKR_66_) and P126 (_35_HNDVYISLPI TLLHFPAHFQ KFSQPAEISDKR_66_). However, the defective-Ig mAb 6D10 minorly reacted with all truncated peptides. This finding is similar to the previous study [49]. The defective-Ig mAbs likely have broad binding, moderate specificity, and low affinity with the associated peptide. There were concerns about this defective-Ig mAb since this seems to be very rare. Further, the molecular weight of the mAb 6D10 was determined by Western blotting. Two bands were observed at about 33 and 6 kDa, respectively. The small one (6 kDa) implied some degradation of this mAb. Since the molecular weight of the light chain was 25 kDa, the author suggested that the mAb 6D10 might contain a short fragment of a protein. Interestingly, the author also tested the molecular weight of another defective-Ig mAb 6C3 [49] (which against peptide C3), and two molecules at about 33 and 6 kDa were again shown (data not shown). According to previous documents, human patients with abnormal serum immunoglobulin-free light chain production should only be due to monoclonal plasmaproliferative disorders (included multiple myeloma, light chain myelomas, and light chain amyloidosis) [51,52,53]. Arguably, the defective-Ig mAb (6D10) might be related to the phenotype of splenocytes, which fused with the myeloma cell. Further research should be carried out using advanced techniques (such as liquid chromatography with mass spectrometry and spectroscopic techniques) to investigate the constitution of the mAb 6D10.

Based on previous reports, lymphocyte depletion and apoptosis in lymphoid tissues are histological hallmarks in PCV2-infected pigs [54,55,56]. Interestingly, these histopathological lesions are similar to Marek’s disease and African swine fever [57,58]. More recent evidence shows that these viruses caused an early cytolytic infection in lymphocytes by apoptosis [57,58,59]. Therefore, this study hypothesized that lymphocyte and PBMCs lineage cells should be the primary target cells for PCV2 infection. To the best of the author’s knowledge, the study of the ORF3 protein by indirect immunofluorescence assay in PCV2-infected PBMCs has never been performed, and only a single article mentions the transient expression of the ORF3 in porcine PBMCs and detecting apoptosis with a TUNEL assay [50]. Therefore, the author explored the relation of the capsid, p53 protein, and the ORF3 protein by IFA and shed light on p53 protein (the marker of apoptosis) accompanied the peptide (residues 35–66, P126) of the ORF3 protein in PCV2-infected PBMCs. It is worth highlighting that 3–5% of PBMCs were positive for some antibodies staining, including anti-ORF3 mAb (7D3) staining, anti-p53 protein rabbit polyclonal antibody staining, and TUNEL assay (Figure S3). There is a strong probability that 3–5% of PBMCs were undergoing the process of apoptosis. However, the data revealed that the variable percentage (4–82%) of PBMCs was positive for anti-capsid mAbs (1H3, 6B8, and 36A9) staining and anti-VLP rabbit serum staining. It was because these mAbs or antiserum recognized different epitopes of proteins presented in PCV2-infected cells. That means that the variety of interaction between antigen-binding sites of antibodies and epitopes, which includes linear form epitopes, conformational epitopes, degradation, and different PCV2 strains. Curiously, this study (Figure S3) showed that the percentage of PCV2-infected PBMCs (by mAb 1H3 staining) was about 20-fold the rate of ORF3-positive PBMCs (by mAb 7D3 staining) or the percentage of p53-positive PBMCs. It seems likely that not all PCV2-infected cells contained ORF3 proteins or p53 protein. This finding concurs with the previous study, which indicated that the apoptosis statistically decreases in the initial PCV2-infected pigs [60].

On the other hand, this study showed the ORF3 protein colocalized with the capsid protein marker of PCV2. Moreover, the p53 protein was also colocalized with the ORF3 protein. Remarkably, the p53/ORF3 dual-positive cell presented a segmented nucleus (Figure 3W). However, these p53/ORF3 dual-positive cells were very few in these samples. This finding will be confirmed by flow cytometry in the future. Although a previous study indicated that PCV2-infected pigs had a reduction of lymphocytes in the peripheral blood [11], the mechanism of lymphocyte lysis was still unclear. This study confirmed that the ORF3 protein was related to the p53 protein (apoptosis marker) in PCV2-infected PBMCs, while other factors (proliferative activity [60], caspases 8 and 3 [34,61], granzymes [62], or corticosteroids [63,64]) causing lymphocyte lysis or depletion need to be considered. Although the experiment on apoptosis in PCV2-infected PBMCs was carried out by the TUNEL assay (Figure 4), it did not clarify the relationship between the TUNEL result and the ORF3 protein in PCV2-infected cells. Only the IFA data confirm previous reports that exogenous ORF3 protein was related to the accumulation of p53 protein [37,42], but more proof are needed to make sure the ORF3 protein is a major factor leading to apoptosis in PCV2-infected cells [34,37,42] and depletion of lymphocytes.

PCV2 is one of the most critical pathogens in modern swine production and causes endemic disease in pig farms [7]. The commercial vaccines were confirmed protective in the field against PCV2 and mainly administered to suckers in herds with PCV2 infection [13,14,65,66,67]. Most researchers utilized the quantitative real-time PCR to detect PCV2 nucleic acid, and then they evaluated the efficacy of vaccination in regard to PCV2 viremia in pigs [13,14,66]. However, little is known about the capsid protein or the ORF3 protein in blood from the PCV2-infected herd with vaccination. Although the capsid antigen-ELISA could detect capsid protein, this assay could not differentiate from native viral proteins or vaccine ones in blood. The ORF3 protein-ELISA, by contrast, only identified native ORF3 proteins in pig blood since no commercial vaccine contains the ORF3 protein of PCV2. For these purposes, this study used the commercial capsid antigen-ELISA and homemade ORF3 protein-ELISA (anti-N1 polyclonal antibodies and mAb 7D3 based) to detect viral proteins in pig blood. This study found non-vaccinated suckers had the highest of PCV2 proteins in blood at one week of age. The author suggests that in these suckers it was caused by PCV2 infection, and these viruses majorly stemmed from the sow-to-newborn transmission [68]. After inoculated with the PCV2 subunit vaccine, the capsid protein, and the ORF3 protein were gradually decreased. However, these proteins were detected again in gilts and sows (≥34 weeks of age). That means PCV2 viral proteins (the capsid protein or the ORF3 protein) in gilts and sows were higher than that of pigs at 9–24 weeks of age. To put it another way, vaccinated-pigs reduced viremia compared to non-vaccinated suckers, and the immunity could not continue to protect vaccinated pigs after 32 weeks post-vaccination. Since PCV2 is highly resistant to environmental conditions, being able to remain in the farm environment and thus represent a risk for infection maintenance [69], even mass PCV2 vaccination (without implementing further farm management practices or biosafety measures) was not able to clear out PCV2 infection, and the virus became detectable again when vaccination was stopped [12,67].

Another key point to mention is that PCV2-specific antibodies response play roles in the PCV2-infected herd. It is worth noting that there were two different antibody profiles in this PCV2-infected herd with the PCV2 vaccine. The data show that PCV2-infected herd had a higher OD_405_ value of anti-capsid IgG at age 1, 3, and ≥12 weeks, compared with 6–9 weeks. In addition, PCV2-infected herd had a higher OD_405_ value of anti-ORF3 IgG at age 1 and ≥9 weeks, compared with 3–6 weeks. According to previous studies, newborn suckers received colostral antibodies from seropositive sows and showed various levels of maternally derived antibodies [48,68]. These maternally derived antibodies might interfere with the viral protein-specific antibodies response while suckers immunized with the PCV2 subunit vaccine. It may be assumed that the artificial capsid protein (PCV2 vaccine) elicited anti-capsid IgG producing while maternally derived antibodies progressed to degrade. The total level of anti-capsid IgG decreased slowly but still maintained a high level of anti-capsid IgG during the age of 1–6 weeks. This phenomenon could neutralize the PCV2 virus and prevent piglets from suffering PCV2 infection. In contrast with dual-source capsid proteins (PCV2 virus and subunit vaccine), the piglet’s immunity response to the ORF3 protein was only caused by PCV2 virus infection. The total level of anti-ORF3 IgG decreased quickly at 1–3 weeks of age. Then, it increased significantly at 6–9 weeks of age. The weak correlation (*r* = 0.3771) between the anti-capsid IgG and the anti-ORF3 IgG is worth mentioning. It might reflect two kinds of antibody responses in virus infection and post-immunization.

These data interpret the interaction of the viral protein with the host immune system. According to previous reports, ORF2 encodes a 30-kDa protein that is involved in viral capsid formation and contributed to self-assembled virus-like particles (VLP) [32]. The monomer structures were assembled into a VLP model consisting of 60 capsid subunits to form an icosahedron [70]. Vaccination with this VLP (subunit vaccine) induced both humoral and cell-mediated responses against PCV2 [71,72]. However, ORF3 encodes an 11.9-kDa protein [28] that is the non-structural protein and involved in PCV2-induced apoptosis [34,37,42]. Until now, no report mentions that the ORF3 protein was solely used as the vaccine against PCV2, reducing viremia or pathological lesions in PCV2-infected herds. These differences contributed to their application in different ways [36,73]. In this study, the percentage of ORF3-positive PBMCs was significantly lower than capsid-positive PBMCs. Similarly, the antigen-ELISA result shows that the amount of ORF3 protein was less than the amount of capsid protein in PCV2-infected blood from suckers at one week of age. That might be because mAb 7D3 only recognizes the ORF3 protein (peptide P126) for PCV2b strain (GenBank: AAC35332.1), and it causes low detection. However, PCV2d (PCV2b mutant strain) and PCV2b are still predominant genotypes in the field farms. Besides the similar percentages of PBMCs were positive for these antibodies (mAb 7D3 and anti-p53 protein rabbit polyclonal antibody) staining or TUNEL assay. In contrast to the ORF3 protein, the detection of the capsid protein could be the variable result in PCV2-infected PBMCs via different antibodies, due to different PCV2 strains [49,74], conformational epitopes [75], or linear epitopes [49,76]. Previous studies indicated that antibody binding residues were often on the exterior of the capsid of PCV2 [48,49,70,74,77,78]. Although an epitope might be on the surface of the single capsid unit, it could bury in the VLP and be inaccessible to the antibody [79,80].

In general, these results confirm that mAb 7D3 recognized the native ORF3 protein in PCV2-infected PBMCs. This study used mAb 7D3 or its minimal linear epitope to design various immunoassays. These assays evaluate the viral load and the immune response of viral proteins stimuli. These may serve as surveillance tools for monitoring natural PCV2 infection in the herd.

## 5. Conclusions

Overall, the author generated anti-ORF3 mAbs against the ORF3 peptide (residues 36–66) of PCV2. This study confirmed the defective-Ig mAb (6D10) with a lambda-light chain, and its molecular weight was about 33 kDa. The mAb 7D3 contained heavy chains (γ_1_) and kappa-light chains, and it bound to one minimal linear epitope (HNDVYISLPITLLHFPAHFQ KFSQPAEISDKR). The data show that 3–5% of PBMCs were positive for ORF3 protein or p53 protein. Otherwise, 78–82% of PBMCs were positive for anti-capsid peptide mAb (1H3) staining. This study confirmed the ORF3 protein colocalized with the p53 protein in PCV2-infected PBMCs. The author devised the ORF3 protein-ELISA (anti-ORF3 antisera and mAb 7D3-based) to detect the ORF3 protein in blood samples. The results show that the amount of ORF3 protein was less than the amount of capsid protein in PCV2-infected blood from suckers at one week of age. The correlation between the ORF3 protein and the capsid protein is worth noting (*r* = 0.7583). Furthermore, the antibody level of anti-capsid IgG and anti- ORF3 IgG could imply the immunity response of pig herd, but the sample size needs to be considered.

## Figures and Tables

**Table 1:** Peptide sequences of the truncated ORF3 protein of PCV2 and negative control peptide (the capsid protein of PCV2b).

Name	PCV Type	Position	Peptide Sequence
N1	2b	ORF3(35–66)	CHNDVYISLPITLLHFPAHFQKFSQPAEISDKR
P31	2b	ORF3(35–44)	HNDVYISLPI
P32	2b	ORF3(45–54)	TLLHFPAHFQ
P33	2b	ORF3(55–66)	KFSQPAEISDKR
P40	2b	ORF3(40–49)	ISLPITLLHF
P41	2b	ORF3(50–59)	PAHFQKFSQP
P42	2b	ORF3(60–68)	AEISDKRRV
P48	2a	ORF3(35–44)	HNDVYIGLPI
P49	2	ORF3(35–44)	HNDVYIRLPI
P56	1	ORF3(35–44)	HYDVYSCLPI
P61	2b	ORF3(35–47)	HNDVYISLPITLL
P69	2b	ORF3(45–66)	TLLHFPAHFQKFSQPAEISDKR
P75	2b	ORF3(40–59)	ISLPITLLHFPAHFQKFSQP
P126	2b	ORF3(35–66)	HNDVYISLPITLLHFPAHFQKFSQPAEISDKR
C3	2b	CP(195–233)	CHVGLGTAFENSIYDQEYNIRVTMYVQFREFNLKDPPLNP

Peptide C3 and peptide N1 were appended with an N-terminal cysteine during synthesis, which was required for conjugation with maleimide-activated carriers.

## Supplementary Materials

The following are available online at https://www.mdpi.com/1999-4915/12/9/961/s1.

## Abbreviations

ABTS	2,2′-Azino-bis(3-ethylbenzothiazoline-6-sulfonic acid) diammonium salt
ANOVA	Analysis of variance
CP	Capsid protein
Cterminus	Carboxyl-terminus
DAPI	4′,6-diamidino-2-phenylindole
DMEM	Dulbecco’s modified Eagle medium
EDTA	Ethylenediaminetetraacetic acid
HRP	Horseradish peroxidase
iELISA	Indirect enzyme-linked immunosorbent assay
IFA	Immunofluorescence assay
Ig	Immunoglobulin
KLH	Keyhole limpet hemocyanin
mAb	Monoclonal antibody
OD	Optical density
ORF	Open reading frame
PBMCs	Peripheral blood mononuclear cells
PBS	Phosphate-buffered saline
PBST	PBS containing 0.05% Tween 20
PCV	Porcine circovirus
PCV1	Porcine circovirus type 1
PCV2	Porcine circovirus type 2
PEG	Polyethylene glycol
pPirh2	Porcine p53-induced RING-H2
RING	Really interesting new gene
SCF	Suspending cells were fixed
SPF	Specific pathogen-free
TRITC	Tetramethyl rhodamine isothiocyanate
TUNEL	Terminal deoxynucleotidyl transferase dUTP nick end labeling
VLP	Virus-like particle
