# Supplementary material for: The Monoclonal Antibody Recognized the Open Reading Frame Protein in Porcine Circovirus Type 2-Infected Peripheral Blood Mononuclear Cells
Source: Viruses. 2020 Aug 29;12(9):961. doi: 10.3390/v12090961 (PMC7551997; doi:10.3390/v12090961)
Supplement: Supplementary file 1 [file viruses-12-00961-s001.pdf]

Supplementary Materials

The monoclonal antibody recognized the ORF3 protein in PCV2-infected peripheral blood mononuclear cells

Ling-Chu Hung [1,2,*,†]

[1] Animal Health Research Institute, Council of Agriculture, Executive Yuan, New Taipei City 25158, Taiwan; lchung@mail.nvri.gov.tw
[2] Livestock Research Institute, Council of Agriculture, Executive Yuan, Tainan 71246, Taiwan;
* Correspondence: lchung@mail.nvri.gov.tw; Tel.: +88-6226-212-111
† Current address: Animal Health Research Institute, Council of Agriculture, Executive Yuan, New Taipei City 25158, Taiwan

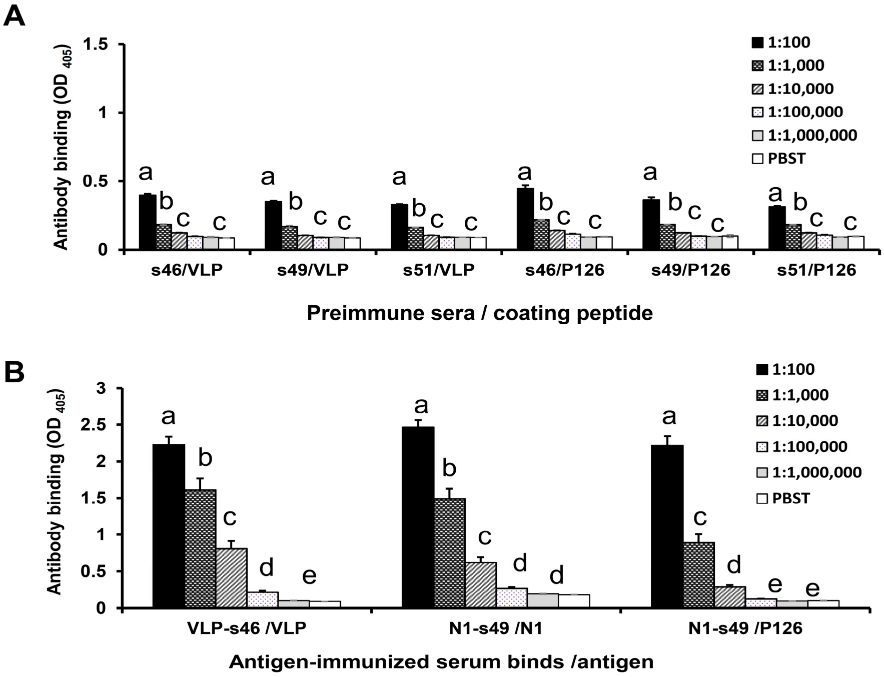

C

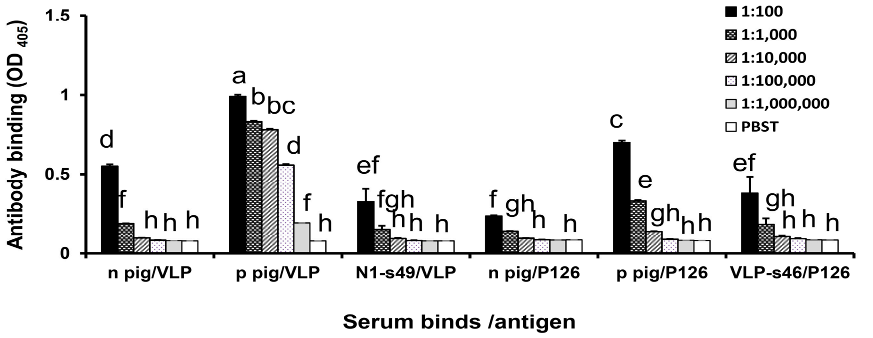

Supplementary Figure S1. Detection of rabbit anti-PCV2 capsid and anti-ORF3 peptide IgG in pre-immunization and post-immunization. The serially diluted sera were applied on the ELISA plate as primary antibodies, and PBS was used as the negative control in each assay. (A) Blood samples were collected from the rabbits (r46, r49, and r51) before immunization, and these serum samples (s46, s49, and s51) were all used to detected anti-VLP and anti-P126 specific antibody by ELISA. Data represent the mean ± SEM. Treatments with different letters have statistically significant differences at that same dilution. (B) Blood samples were collected from the rabbits (r46 and r49) after five inoculations (r46 immunized with VLP, and r49 immunized with N1), and these serum samples (VLP-s46, and N1-s49) were used to detected anti-VLP, anti-P126, and anti-N1 specific antibody by ELISA. Data represent the mean ± SE and are representative of four independent assays, each with two replicate wells. Treatments with different letters have statistically significant differences at that same dilution. (C) The PCV2-negative serum (n pig, a three-month-old pig) was collected from the PCV2-negative herd in the primary SPF pig facility. The PCV2-positive serum (p pig, a 21-week old pig) was collected from the unvaccinated naturally PCV2-infected pig farm. These serum samples (n pig, p pig, N1-s49, and VLP-s46) were used to detected anti-VLP and anti-P126 specific antibodies by ELISA. Data represent the mean ± SE. The experiments were performed at least twice, each with two replicate wells. Treatments with different letters have statistically significant differences at that same dilution.

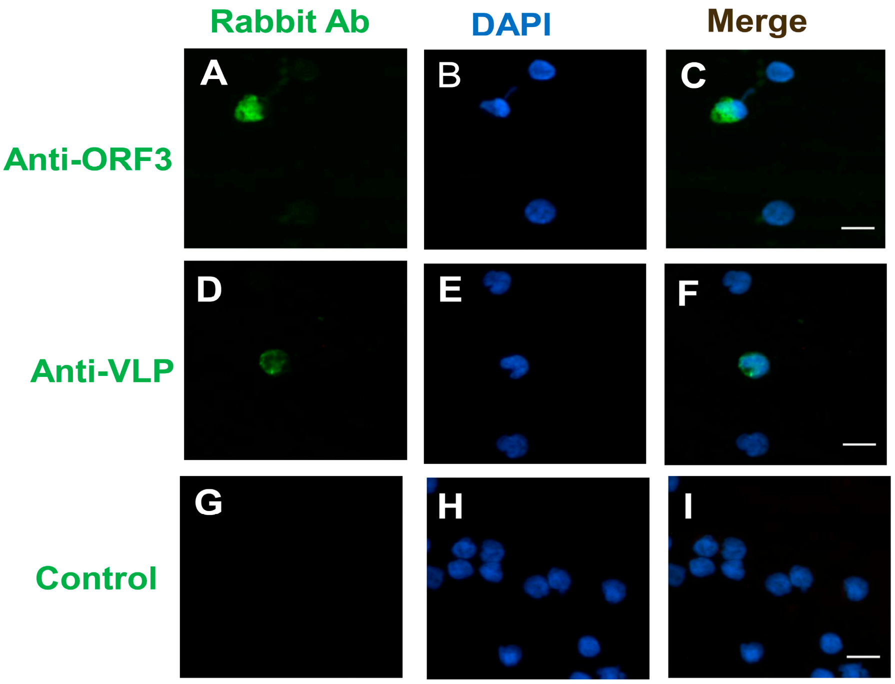

Supplementary Figure S2. The localization of viral proteins of PCV2 by indirect IFA. PCV2-infected PBMC were stained with anti-ORF3 (the peptide N1) rabbit serum, anti-capsid (VLP of PCV2) rabbit serum, and pre-immune (negative control) rabbit serum, respectively. Left column (A, D, and G): Fluorescence microscopy of viral proteins of PCV2 were identified (green). Middle column (B, E, and H): Nuclei were stained with DAPI (blue). Right column (C, F, and I): the merge of the images is shown. The scale bar represents 10 μm.

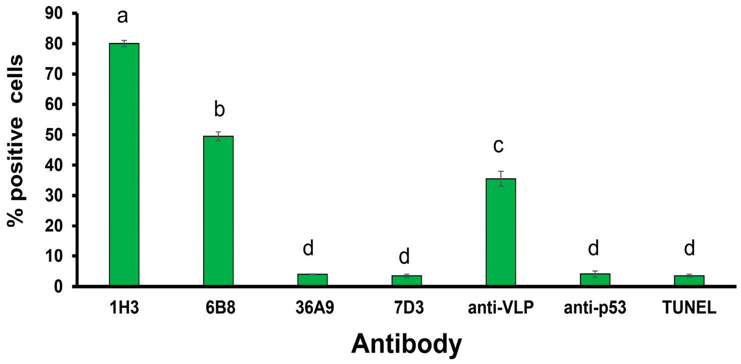

Supplementary Figure S3. Staining of the capsid protein of PCV2, ORF3 protein, p53 protein, and DNA breakage in PCV2-infected PBMCs by different antibody or assay. PCV2-infected PBMC were stained with anti-capsid protein mAbs (1H3, 6B8, and 36A9), anti-ORF3 protein mAb (7D3), anti-capsid (VLP of PCV2) rabbit serum, anti-p53 protein rabbit polyclonal antibody, and TUNEL assay, respectively. The percentage was determined by counting the number of positive cells per 100 nuclei in an IFA slide. Data represent the mean ± SD. Treatments with different letters have statistically significant differences.

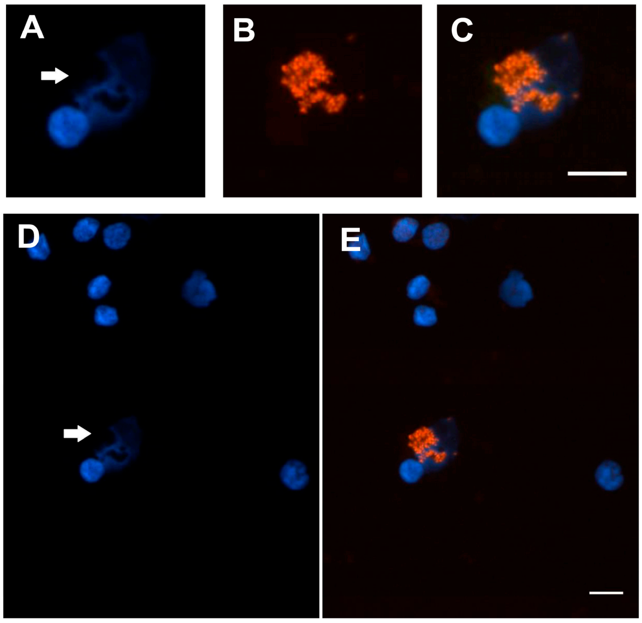

Supplementary Figure S4. Detecting excessive DNA breakage in the nuclear rupture of PBMC (positive control). The Cell Meter™ TUNEL Apoptosis Assay kit was performed according to the manufacture's instruction. Left column (**A** and **D**): Nuclei were stained with Hoechst, the arrow pointed to a rupture of the nucleus (**D**), and the enlarged image in the upper left panel (**A**). Staining with TF3-dUTP /reaction buffer (red) was used to detect DNA fragmentation (**B**). The merge of the images is shown (**C** and **E**). The scale bar is 10 μm.

.
